# Supplementary material for: Genetic diversity, determinants, and dissemination of Burkholderia pseudomallei lineages implicated in melioidosis in Northeast Thailand
Source: Nat Commun. 2024 Jul 7;15:5699. doi: 10.1038/s41467-024-50067-9 (PMC11228029; doi:10.1038/s41467-024-50067-9)
Supplement: Supplementary file 1 — Supplementary Information [file 41467_2024_50067_MOESM1_ESM.pdf]

Supplementary Information

Genetic diversity, determinants, and dissemination of *Burkholderia pseudomallei* lineages implicated in melioidosis in northeast Thailand

Rathanin Seng, Chalita Chomkatekaew, Sarunporn Tandhavanant, Natnaree Saiprom, Rungnapa Phunpang, Janjira Thaipadungpanit, Elizabeth M Batty, Nicholas PJ Day, Wasun Chantratita, T. Eoin West, Nicholas R Thomson, Julian Parkhill, Claire Chewapreecha*, Narisara Chantratita*

*Contributed equally

Corresponding Authors: Claire Chewapreecha (claire@tropmedres.ac) and Narisara Chantratita (narisara@tropmedres.ac)

This document provides information for Supplementary figure 1-8.

Supplementary Figures

Supplementary Figure 1

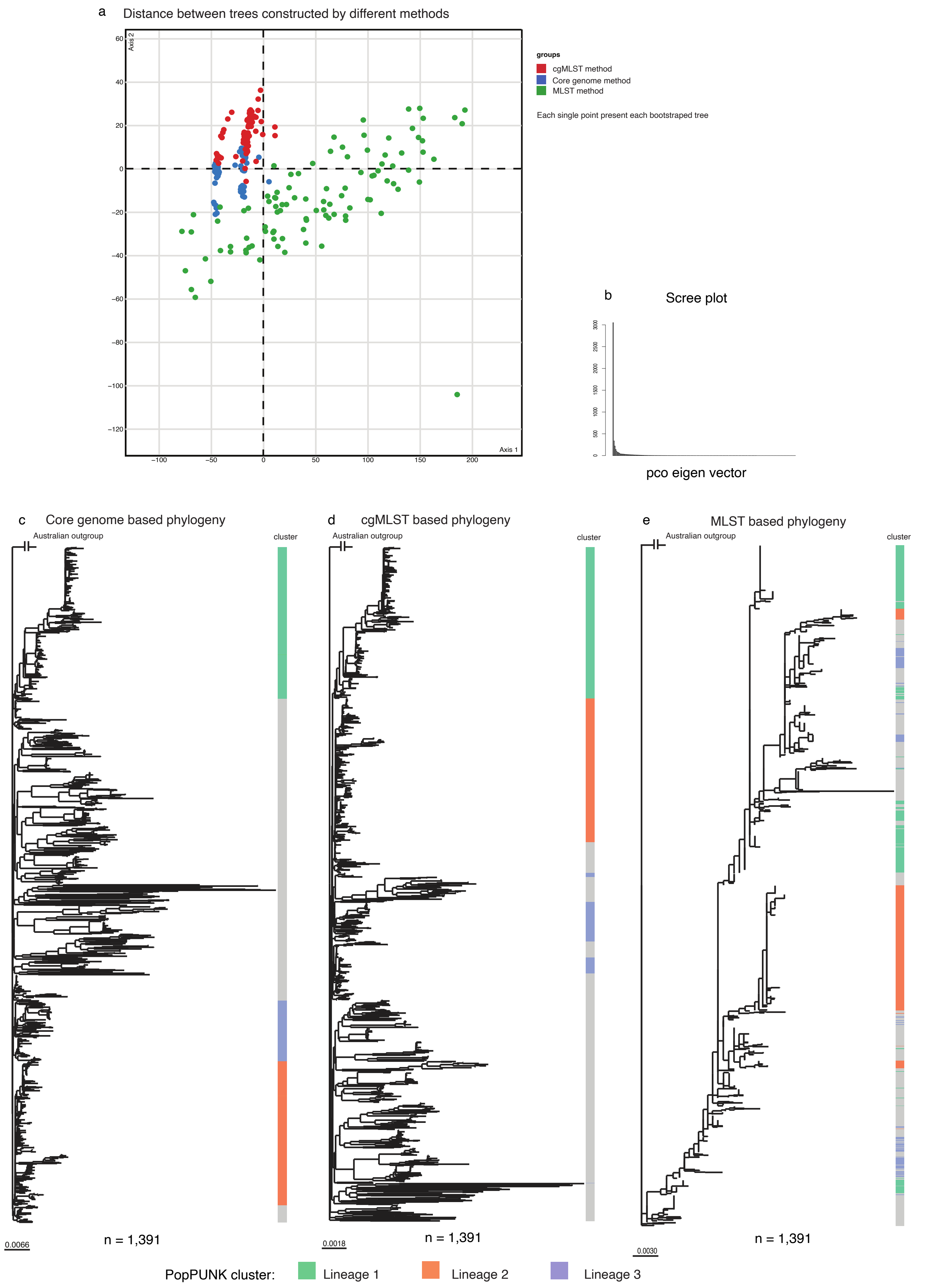

Supplementary Figure 1. Comparison of approaches used in in outlining the population structure (a) A scatter plot displays the first two principal components (PCs) derived from pairwise distances between trees. Each dot represents a bootstrap tree and is colour-coded by the method used to generate the tree: core genome SNP (blue), cgMLST (red) and MLST (green). (b) A scree plot summarises eigenvalues computed for each PCs. (c to e) The median phylogenetic trees constructed from core genome SNP, cgMLST, and MLST and their consistency with PopPUNK clustering method.

Supplementary Figure 2

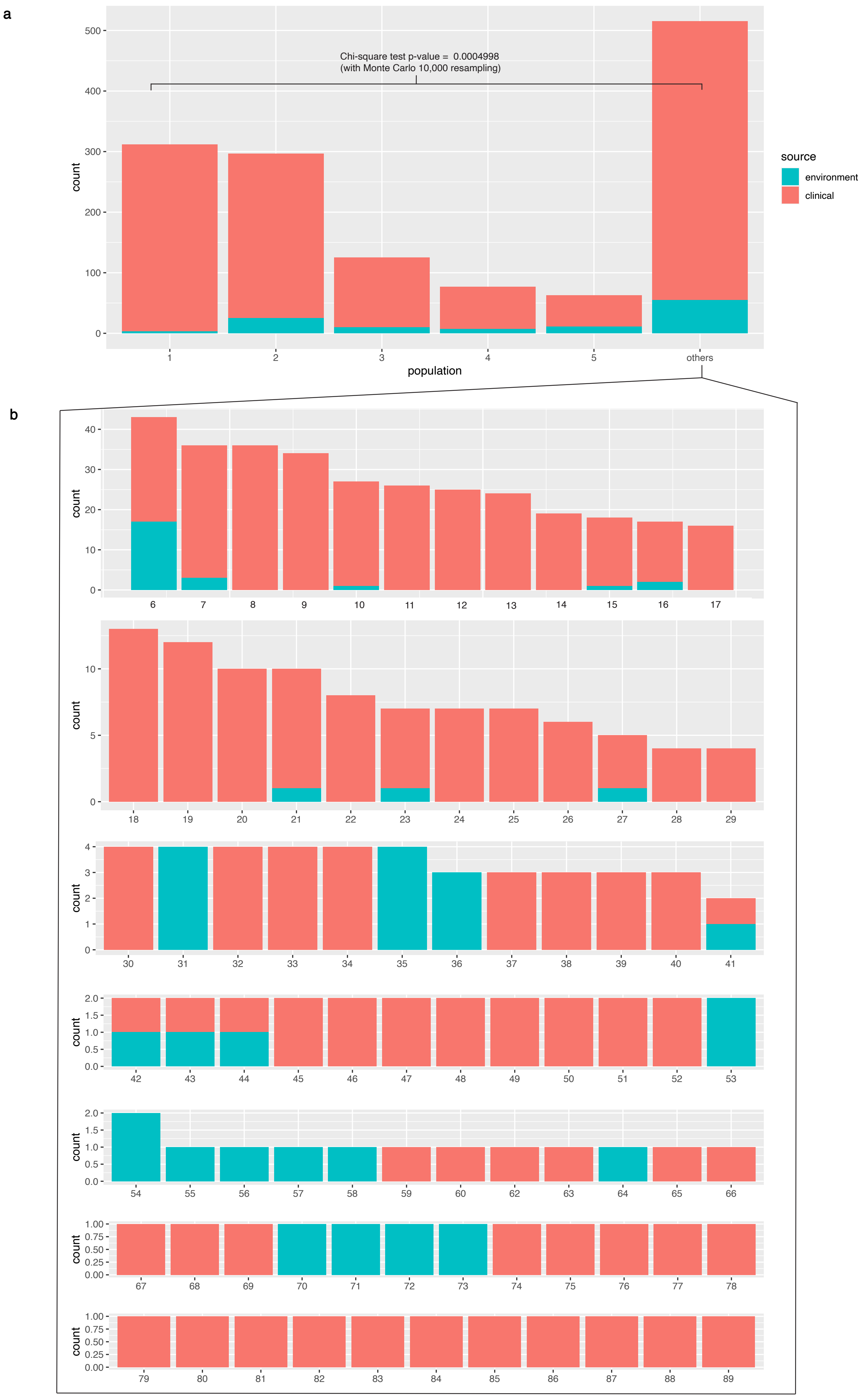

Supplementary Figure 2. Distribution of environmental and clinical isolates by each lineage.

(a) summarises the distribution of isolate origins within the larger lineages: n = 312 (lineage 1), n = 297 (lineage 2), n = 125 (lineage 3), n = 77 (lineage 4), and n = 63 (lineage 5). The remaining isolates are grouped together as "others". The proportions of environmental and clinical isolates are compared using a two-sided Chi-sequare test, with resampling 10,000 times using the Monte Carlo method to account for the lower availability of environmental isolates. (b) represents the distribution of isolates origins for the smaller lineages. In both (a) and (b), the bar plots highlight the co-detection of environmental (green) and clinical isolates (red) across each defined lineage.

Supplementary Figure 3

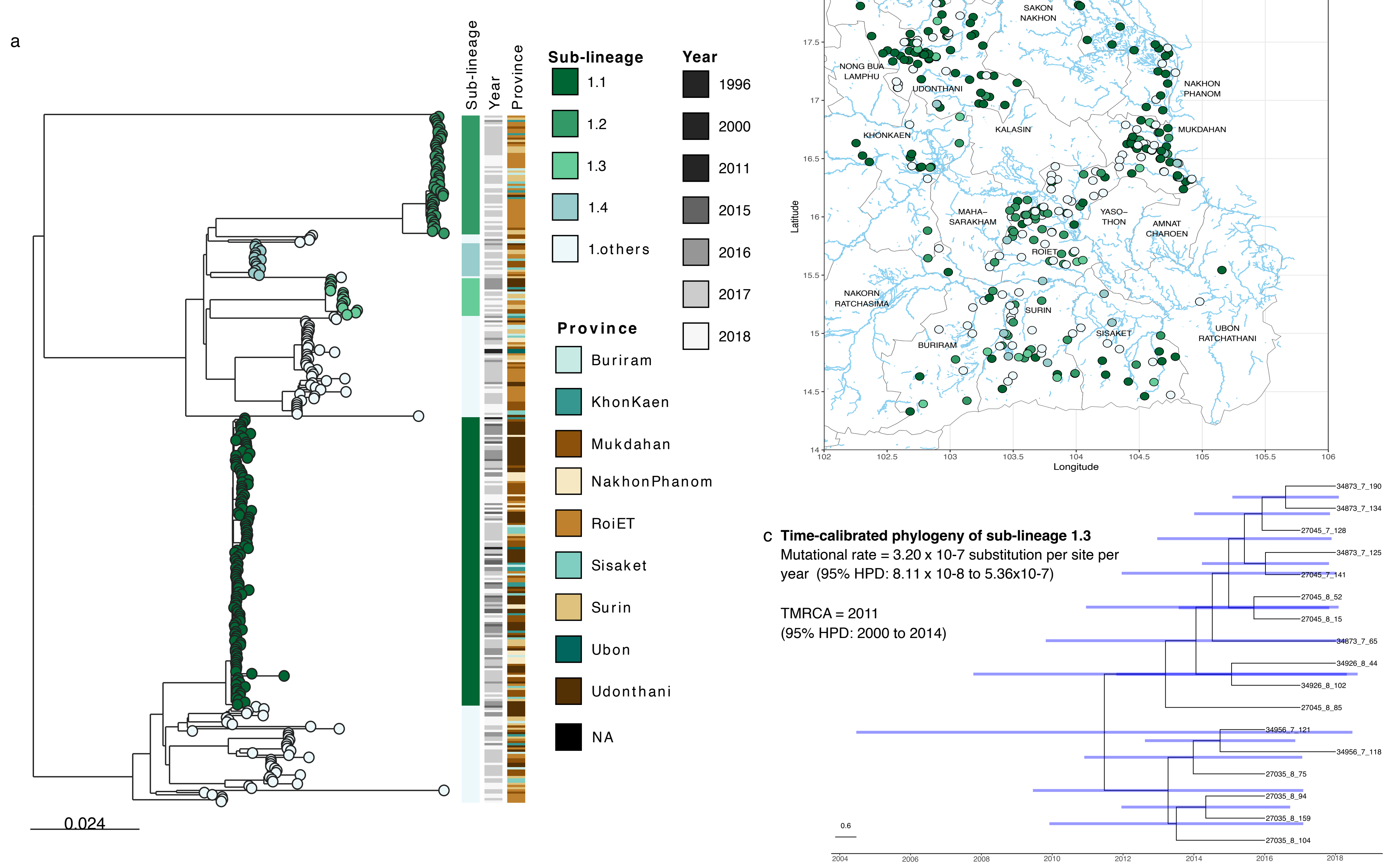

Supplementary Figure 3. Lineage 1 specific analysis (a) A recombination removed lineage 1 phylogeny with colour stripes displaying its sub-lineage structure, year of collection, and sampling province (left to right). (b) A map of northeast Thailand showing the distribution of each isolate and the region's river system. (c) Time-calibrated phylogeny of sub-lineage 1.3 with blue error bars indicating 95% highest posterior density interval, with the estimated mutational rate consistent with previous study.

Supplementary Figure 4

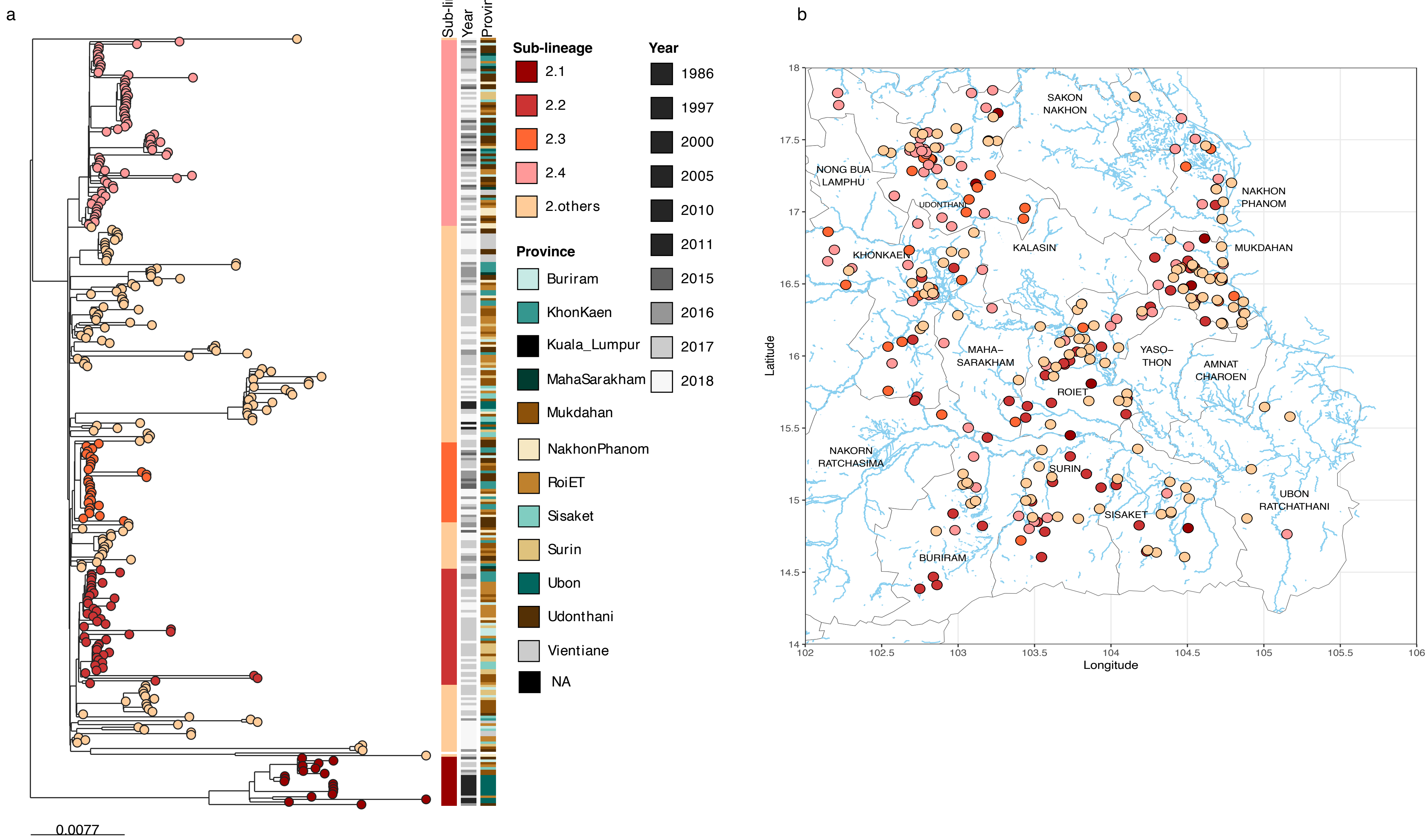

Supplementary Figure 4. Lineage 2 specific analysis (a) A recombination removed lineage 2 phylogeny with colour stripes highlighting sub-lineage structure, year of collection, and sampling province (left to right). (b) A map of northeast Thailand with the region's river system. Dots present the distribution of individual samples.

Supplementary Figure 5

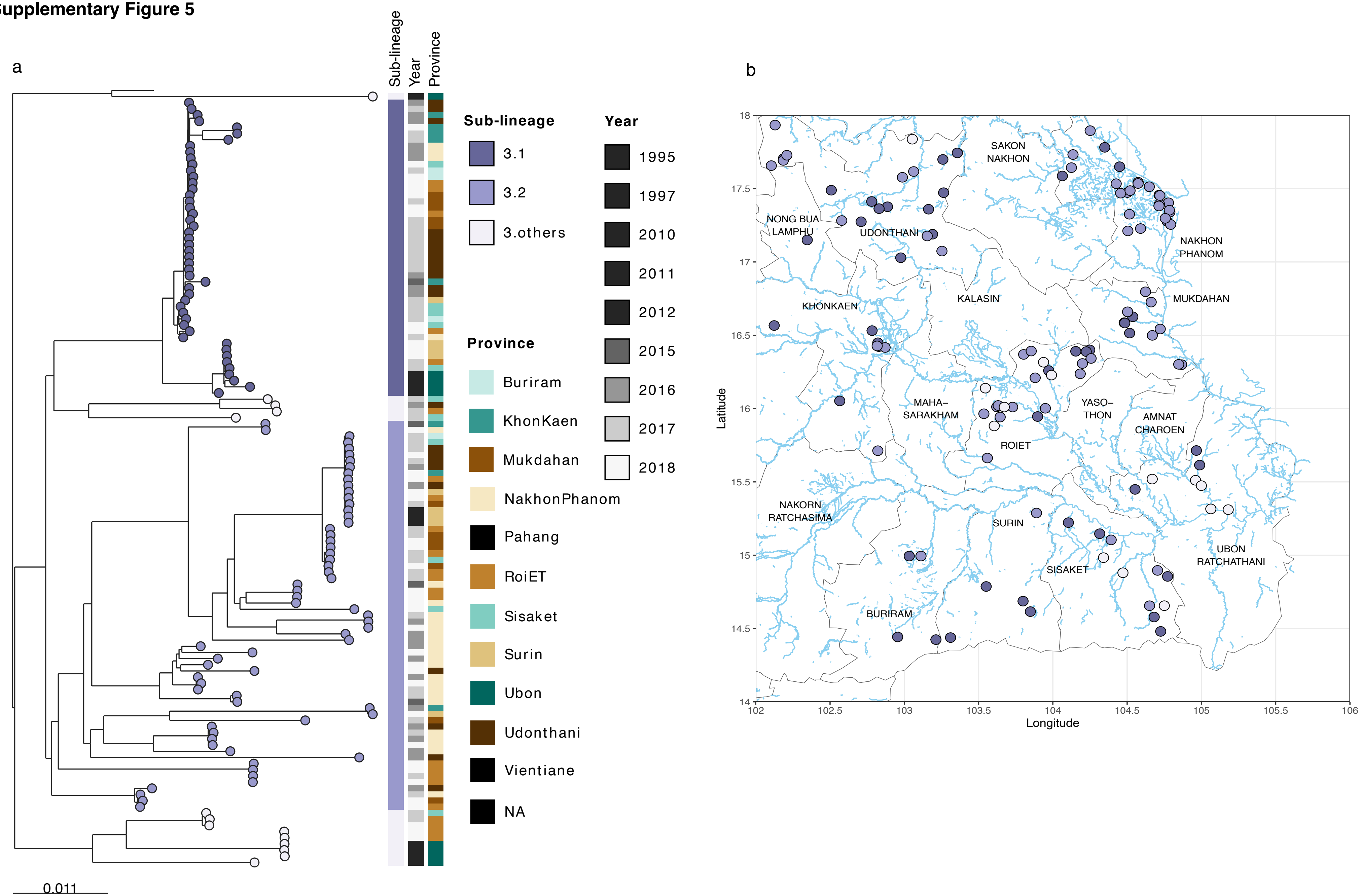

Supplementary Figure 5. Lineage 3 specific analysis (a) A recombination removed lineage 3 phylogeny with colour stripes highlighting sub-lineage structure, year of collection, and sampling province (left to right). (b) A map of northeast Thailand showing the distribution of each isolate and the region's river system.

Supplementary Figure 6

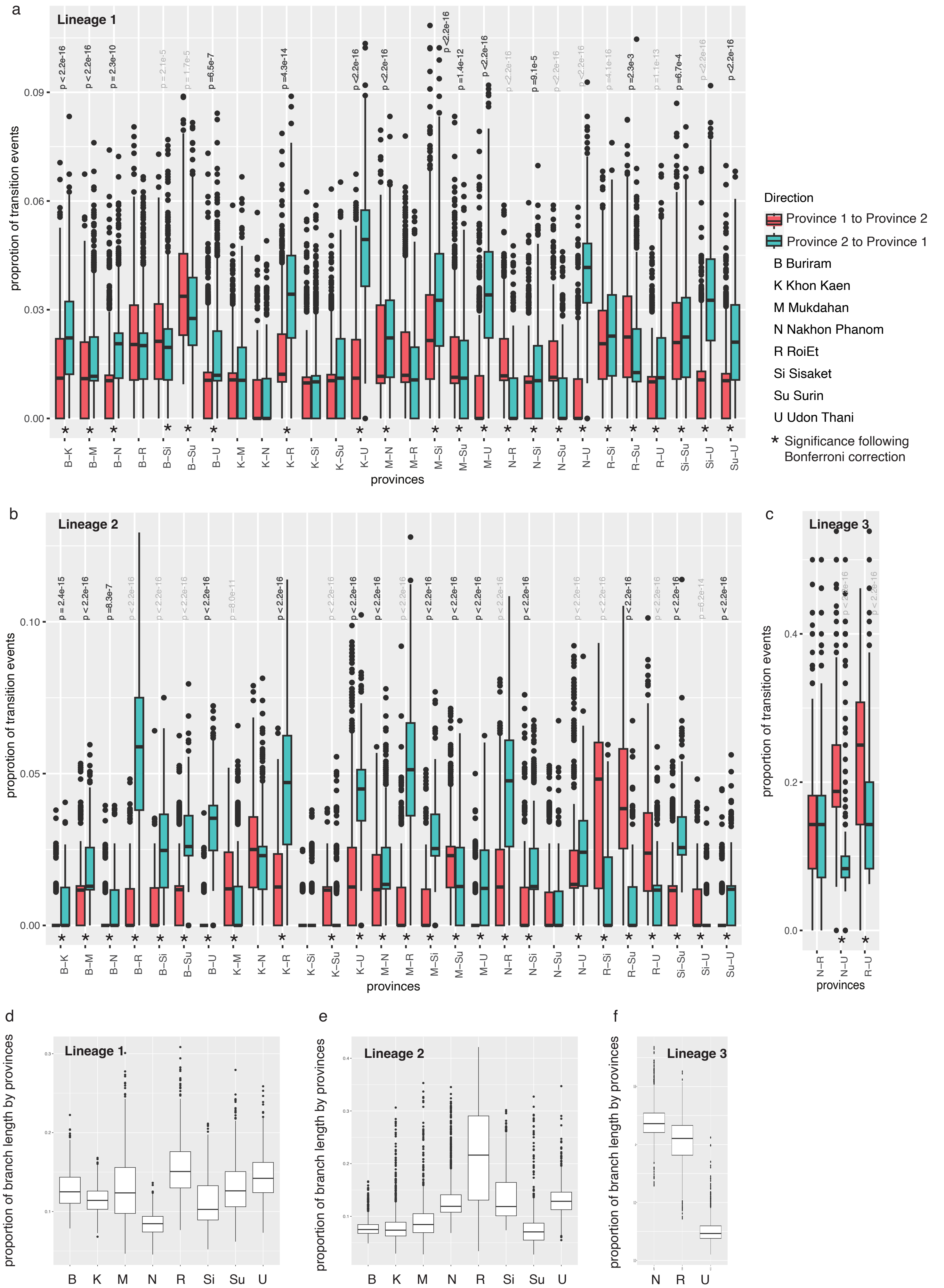

Supplementary Figure 6. Transmission patterns and evolutionary time spent at each province.

(a to c) Panels show the proportion of transition events (Markov jumps) among provincial pairs for lineages 1 (a), 2 (b), and 3 (c), respectively. Each boxplot was generated from 1,000 data points, representing each province-to-province transitions in each subsampled tree (1,000 trees in total, with 100 simulations for each tree). The pairs are denoted as province 1 - province 2, with transitions from province 1 to province 2 shown in red, and transitions from province 2 to province 1 shown in green. A two-sided Mann-Whitney U test was conducted for each pair to assess differences in transition frequency by direction, with Bonferroni correction applied for multiple testings. (d to f) Panels display the total branch length from provincial trait reconstruction (Markov rewards) for lineages 1 (d), 2 (e), and 3 (f), respectively. Each boxplot was generated from 1,000 data points, representing the sum branch-length of each province in 1,000 subsampled trees. Each boxplot presents the minimum, first quartile, median, third quartile, and maximum data points. Each province was abbreviated as follows: U-UdonThani, K-KhonKaen, B-Buriram, R -Roi Et, N-Nakhon Phanom, Su-Surin, M-Mukdahan, and Si-Sisaket. P-values were annotated on figures for provincial pairs with unequal transitions. Black annotations highlight consistent directionality observed in at least two lineages, while grey annotations represent significant patterns unique to one lineage or with contradicting patterns across lineages.

Supplementary Figure 7

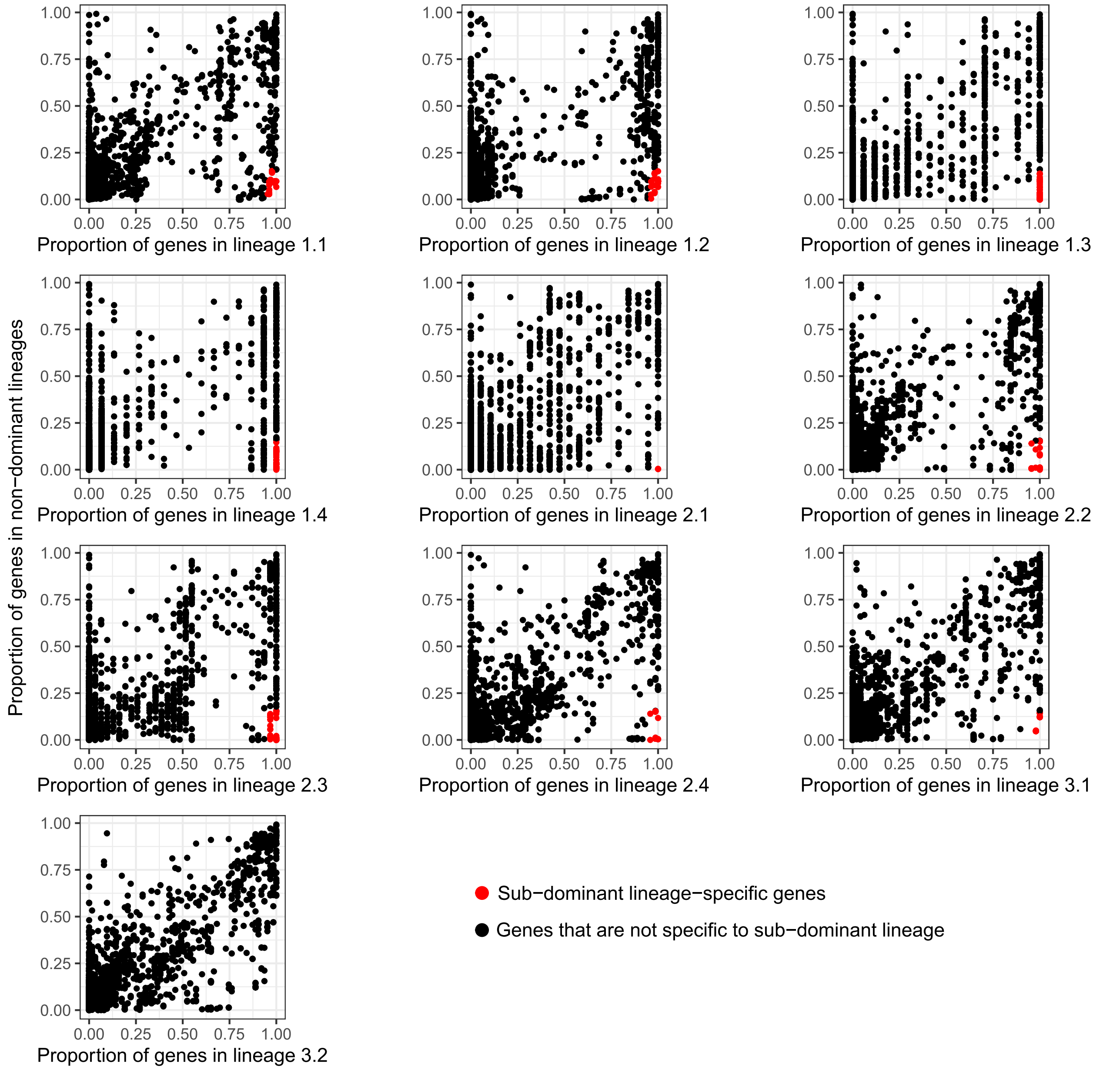

Supplementary Figure 7. Selection criteria for lineage-specific genes. Scatter plots show the frequency distribution of lineage-specific (red) against other genes (black), based on their distribution within the dominant lineages and their sub-lineages (horizontal axis) compared to their distribution in non-dominant lineages.

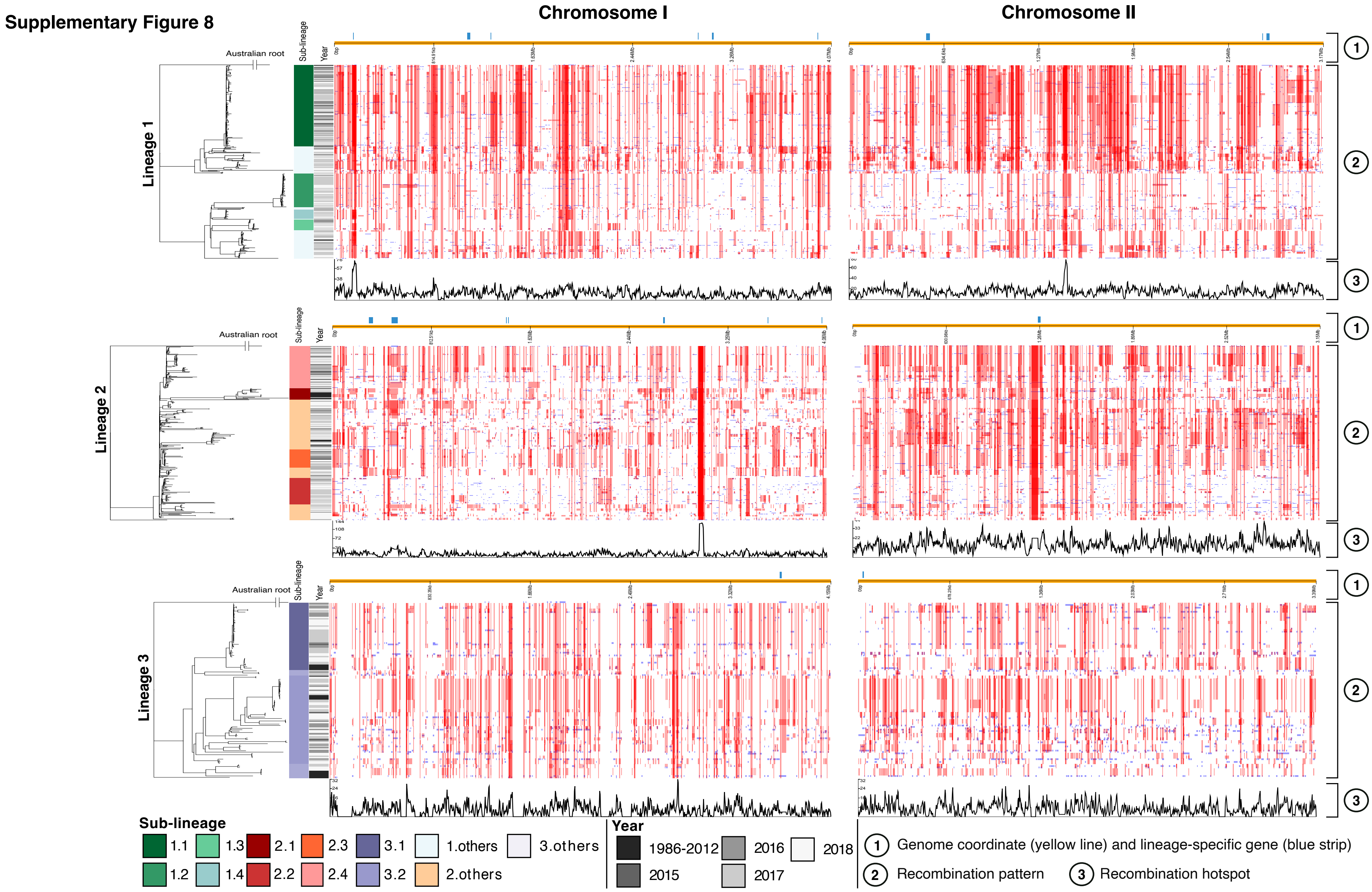

Supplementary Figure 8. Recombination patterns detected in lineage 1, 2, and 3. From left to right: the recombination-removed phylogeny of each lineage, a stripe representing the sub-lineage classification and sampling year, and heatmaps displaying recombination patterns identified in chromosome 1 and 2. The top orange lines mark the genome coordinates. For each lineage, their respective lineage-specific genes are highlighted in blue at the top of the panel. Each heatmap represents recombination blocks aligned with the phylogeny. Recombination events occurring at the internal nodes are coloured in red, while those occurring at the external branches are coloured in blue. The recombination hotspot is plotted at the bottom of each heatmap.
