# Supplementary material for: Genetic diversity, determinants, and dissemination of Burkholderia pseudomallei lineages implicated in melioidosis in Northeast Thailand
Source: Nat Commun. 2024 Jul 7;15:5699. doi: 10.1038/s41467-024-50067-9 (PMC11228029; doi:10.1038/s41467-024-50067-9)
Supplement: Supplementary file 3 — Description Of Additional Supplementary File [file 41467_2024_50067_MOESM3_ESM.pdf]

Description of Additional supplementary file

Supplementary data 1 Epidemiological data, isolate and accession codes for both short reads and annotated assembly used in this study deposited in the European Nucleotide Archive (ENA) (n = 1,391) (provided as a separate excel file).

Supplementary data 2 Quality control information of short-read mapping and assembly of newly sequenced B. pseudomallei from northeast Thailand collection (n = 1,265) (provided as a separate excel file).

Supplementary data 3 List of dominant lineagespecific genes and their Gene Ontology (GO) terms (provided as a separate excel file).

Supplementary data 4 List of dominant lineagespecific genes and their expression profile under infectious and environmental conditions generated by Ooi et al. 2013, Ghazali et al. 2023 and Kong et al. 2023 (provided as a separate excel file).
