# Supplementary material for: Genetic diversity, determinants, and dissemination of Burkholderia pseudomallei lineages implicated in melioidosis in Northeast Thailand
Source: Nat Commun. 2024 Jul 7;15:5699. doi: 10.1038/s41467-024-50067-9 (PMC11228029; doi:10.1038/s41467-024-50067-9)
Supplement: Supplementary file 8 — Reporting summary [file 41467_2024_50067_MOESM8_ESM.pdf]

nature portfolio

Corresponding author(s): Chewapreecha and Chantratita

Last updated by author(s): Jun 12, 2024

# Reporting Summary

Nature Portfolio wishes to improve the reproducibility of the work that we publish. This form provides structure for consistency and transparency in reporting. For further information on Nature Portfolio policies, see our Editorial Policies and the Editorial Policy Checklist.

## Statistics

For all statistical analyses, confirm that the following items are present in the figure legend, table legend, main text, or Methods section.

| n/a | Confirmed | |
|---|---|---|
| ☐ | ☒ | The exact sample size (*n*) for each experimental group/condition, given as a discrete number and unit of measurement |
| ☐ | ☒ | A statement on whether measurements were taken from distinct samples or whether the same sample was measured repeatedly |
| ☐ | ☒ | The statistical test(s) used AND whether they are one- or two-sided<br>*Only common tests should be described solely by name; describe more complex techniques in the Methods section.* |
| ☒ | ☐ | A description of all covariates tested |
| ☐ | ☒ | A description of any assumptions or corrections, such as tests of normality and adjustment for multiple comparisons |
| ☐ | ☒ | A full description of the statistical parameters including central tendency (e.g. means) or other basic estimates (e.g. regression coefficient) AND variation (e.g. standard deviation) or associated estimates of uncertainty (e.g. confidence intervals) |
| ☐ | ☒ | For null hypothesis testing, the test statistic (e.g. *F*, *t*, *r*) with confidence intervals, effect sizes, degrees of freedom and *P* value noted<br>*Give P values as exact values whenever suitable.* |
| ☐ | ☒ | For Bayesian analysis, information on the choice of priors and Markov chain Monte Carlo settings |
| ☒ | ☐ | For hierarchical and complex designs, identification of the appropriate level for tests and full reporting of outcomes |
| ☒ | ☐ | Estimates of effect sizes (e.g. Cohen's *d*, Pearson's *r*), indicating how they were calculated |

*Our web collection on statistics for biologists contains articles on many of the points above.*

## Software and code

Policy information about availability of computer code

| | |
|---|---|
| Data collection | Public genomes were downloaded using SRA Toolkit 2.10.6 using the accession number tabulated in Supplementary data 1 |
| Data analysis | All software utilised for data analysis is open source including: Kraken v.1.1.1, CheckM v.1.2.2, FastANI v.1.31, Velvet v.1.2.10, Snippy v.4.6.0, PopPUNK v.2.6.0, IQ-TREE v.2.0.3, treespace v.1.1.4.3, Unicycler v.0.8.4, ABACAS v.1.3.1, Gubbins v.3.1.3, rhierbaps v.1.1.4, BactDating v.1.1.1, ggtree v.3.10.0, phytools v.1.9.16, Prokka v.1.14.5, Panaroo v.1.3.3, GEOquery v.2.58.0, limma v.3.58.1, FastQC v.0.11.9, FastXtool v.0.0.14, Hisat2 v.2.2.1, DESeq2 v.1.40.2. |

For manuscripts utilizing custom algorithms or software that are central to the research but not yet described in published literature, software must be made available to editors and reviewers. We strongly encourage code deposition in a community repository (e.g. GitHub). See the Nature Portfolio guidelines for submitting code & software for further information.

## Data

Policy information about availability of data

All manuscripts must include a data availability statement. This statement should provide the following information, where applicable:

- Accession codes, unique identifiers, or web links for publicly available datasets
- A description of any restrictions on data availability
- For clinical datasets or third party data, please ensure that the statement adheres to our policy

The newly sequenced 1,265 B. pseudomallei genomes from northeast Thailand generated in this study have been deposited in the European Nucleotide Archive

(ENA) under study accession number PRJEB25606 [https://www.ncbi.nlm.nih.gov/bioproject/?term=PRJEB25606] and PRJEB35787 [https://www.ncbi.nlm.nih.gov/bioproject/?term=PRJEB35787]. The accession numbers for individual genomes are provided in Supplementary Data 1. We sourced existing RNA data used to compare gene expression during infection and in the environment for representative strains of lineages 1, 2, and 3 from the following repositories: NCBI Gene Expression Omnibus (GEO) under accession number GSE43205 (lineage 1) [ https://www.ncbi.nlm.nih.gov/geo/query/acc.cgi?acc=GSE43205], and the ENA under accession numbers E-MTAB-11200 (lineage 2)[ https://www.ebi.ac.uk/biostudies/arrayexpress/studies/E-MTAB-11200] and PRJEB53338 (lineage 3)[ [https://www.ncbi.nlm.nih.gov/bioproject/?term=PRJEB53338]. Source data are provided with this paper. There is no restrictions on data availability.

## Research involving human participants, their data, or biological material

Policy information about studies with human participants or human data. See also policy information about sex, gender (identity/presentation), and sexual orientation and race, ethnicity and racism.

| | |
|---|---|
| Reporting on sex and gender | This information is not reported in the manuscript. |
| Reporting on race, ethnicity, or other socially relevant groupings | This information is not reported in the manuscript. |
| Population characteristics | Patients infected with melioidosis resided in northeast Thailand and likely acquired B. pseudomallei from this endemic region. All melioidosis patients were at least 15 years of age. |
| Recruitment | Patients with microbiologically-confirmed melioidosis were recruited to a cohort described in ref 8. Informed consent was obtained from participants or their surrogate decision-maker. Clinical data and household location were abstracted from the medical record and from patient or surrogate decision-maker interview. |
| Ethics oversight | The ethics committees of each of the nine study hospitals and the Mahidol University Faculty of Tropical Medicine approved the study (approval number MUTM 2015-002-01 and MUTM 2021-055-01). |

Note that full information on the approval of the study protocol must also be provided in the manuscript.

# Field-specific reporting

Please select the one below that is the best fit for your research. If you are not sure, read the appropriate sections before making your selection.

☐ Life sciences ☐ Behavioural & social sciences ☒ Ecological, evolutionary & environmental sciences

For a reference copy of the document with all sections, see nature.com/documents/nr-reporting-summary-flat.pdf

# Ecological, evolutionary & environmental sciences study design

All studies must disclose on these points even when the disclosure is negative.

| | |
|---|---|
| Study description | We analysed 1,391 B. pseudomallei isolates from northeast Thailand and neighbouring countries. We used independent approaches to outline population structure and grouped them into lineages. Subgroup analyses focused on three dominant lineages, excluding smaller groups from the subsequent analyses. These analyses included reconstructing the spread of each lineage geographically over time, identifying genes specific to each lineage and their recombination patterns, and comparing how these genes are expressed in different conditions. Our findings provide insight into how B. pseudomallei persists and spreads in the hyper-endemic region for melioidosis. |
| Research sample | The newly sequenced genomes (n = 1,265) used in this study was extracted from clinical B. pseudomallei isolates collected during our previous longitudinal cohort study in Northeast Thailand (ref 8). The second set of genomes (n = 126) was complied from previously published sources to contextualise our finding. |
| Sampling strategy | In our previous longitudinal cohort study of melioidosis at nine hospitals in Northeast Thailand (ref 8), we screened 2,574 hospitalised culture-confirmed melioidosis cases and 1,372 patients were enrolled. Twenty patients did not meet the enrollment criteria or had delayed enrollment so they were withdrawn. Therefore, 1,352 patients were analysed to define current characteristics and outcomes of melioidosis. In the present study, we sequenced the genomes of 1,282 clinical B. pseudomallei isolates, excluding 17 genomes due to contaminations and low quality reads, Therefore, a total of 1,265 genomes were subjected to analysis. The comprehensive sampling approach provided opportunities to characterise the population structure, dissemination dynamics, and identification of genetic determinants associated with dominant B. pseudomallei lineages in endemic regions. |
| Data collection | For the newly sequenced data, the bacterial data was obtained from the hospital system and then linked to the metadata collected as part of the study in ref 8. |
| Timing and spatial scale | Bacterial isolates were systematically collected form the years 1986 to 2018, with the following distributions: 1986 = 1; 1995 = 1; 1996 = 6; 1997 = 9; 2000 = 9; 2002 = 1; 2005 = 1; 2010 = 13; 2011 = 33; 2012 = 2; 2015 = 62; 2016 = 217; 2017 = 596; 2018 = 437; and NA = 3. The collection spans diverse geographic location, including Northeast Thailand (n = 1,318), Laos (n = 40), Malaysia (n = 14), Myanmar (n = 7), and Singapore (n = 12). |
| Data exclusions | Genomes with contaminations and low quality reads were excluded. |

| | |
|---|---|
| Reproducibility | We employed independent approaches to define bacterial population structure, ensuring reproducible results. |
| Randomization | Date-randomisation permutation tests were performed to ensure that the detected temporal signals were not random. Additionally, permutations were conducted to reduce bias from the varying number of isolates from each province during the reconstruction of province-to-province dissemination history |
| Blinding | The investigators were not blinded to group allocation as the outcomes were objectively measured and did not require subjective interpretation. |

Did the study involve field work? ☐ Yes ☒ No

# Reporting for specific materials, systems and methods

We require information from authors about some types of materials, experimental systems and methods used in many studies. Here, indicate whether each material, system or method listed is relevant to your study. If you are not sure if a list item applies to your research, read the appropriate section before selecting a response.

## Materials & experimental systems

| n/a | Involved in the study |
|---|---|
| ☒ | ☐ Antibodies |
| ☒ | ☐ Eukaryotic cell lines |
| ☒ | ☐ Palaeontology and archaeology |
| ☒ | ☐ Animals and other organisms |
| ☒ | ☐ Clinical data |
| ☒ | ☐ Dual use research of concern |
| ☒ | ☐ Plants |

## Methods

| n/a | Involved in the study |
|---|---|
| ☒ | ☐ ChIP-seq |
| ☒ | ☐ Flow cytometry |
| ☒ | ☐ MRI-based neuroimaging |

## Plants

| | |
|---|---|
| Seed stocks | *Report on the source of all seed stocks or other plant material used. If applicable, state the seed stock centre and catalogue number. If plant specimens were collected from the field, describe the collection location, date and sampling procedures.* |
| Novel plant genotypes | *Describe the methods by which all novel plant genotypes were produced. This includes those generated by transgenic approaches, gene editing, chemical/radiation-based mutagenesis and hybridization. For transgenic lines, describe the transformation method, the number of independent lines analyzed and the generation upon which experiments were performed. For gene-edited lines, describe the editor used, the endogenous sequence targeted for editing, the targeting guide RNA sequence (if applicable) and how the editor was applied.* |
| Authentication | *Describe any authentication procedures for each seed stock used or novel genotype generated. Describe any experiments used to assess the effect of a mutation and, where applicable, how potential secondary effects (e.g. second site T-DNA insertions, mosiacism, off-target gene editing) were examined.* |
